# Lactic Acid Transport Mediated by Aquaporin-9: Implications on the Pathophysiology of Preeclampsia

**DOI:** 10.3389/fphys.2021.774095

**Published:** 2021-12-02

**Authors:** Yollyseth Medina, Lucas Acosta, Julieta Reppetti, Ana Corominas, Juanita Bustamante, Natalia Szpilbarg, Alicia E. Damiano

**Affiliations:** ^1^Laboratorio de Biología de la Reproducción, Instituto de Fisiología y Biofísica Bernardo Houssay (IFIBIO)- CONICET- Facultad de Medicina, Universidad de Buenos Aires, Buenos Aires, Argentina; ^2^Centro de Altos Estudios en Ciencias Humanas y de la Salud (CAECIHS), Universidad Abierta Interamericana, Buenos Aires, Argentina; ^3^Hospital Nacional Prof. A Posadas, Buenos Aires, Argentina; ^4^Cátedra de Biología Celular y Molecular, Departamento de Ciencias Biológicas, Facultad de Farmacia y Bioquímica, Universidad de Buenos Aires, Buenos Aires, Argentina

**Keywords:** AQP9, lactic acid transport, mitochondria, human placenta, preeclampsia

## Abstract

Aquaporin-9 (AQP9) expression is significantly increased in preeclamptic placentas. Since feto-maternal water transfer is not altered in preeclampsia, the main role of AQP9 in human placenta is unclear. Given that AQP9 is also a metabolite channel, we aimed to evaluate the participation of AQP9 in lactate transfer across the human placenta. Explants from normal term placentas were cultured in low glucose medium with or without L-lactic acid and in the presence and absence of AQP9 blockers (0.3 mM HgCl_2_ or 0.5 mM Phloretin). Cell viability was assessed by 3-(4,5-dimethylthiazol-2-yl)-2,5-diphenyl tetrazolium bromide assay and lactate dehydrogenase release. Apoptotic indexes were analyzed by Bax/Bcl-2 ratio and Terminal Deoxynucleotidyltransferase-Mediated dUTP Nick-End Labeling assay. Heavy/large and light/small mitochondrial subpopulations were obtained by differential centrifugation, and AQP9 expression was detected by Western blot. We found that apoptosis was induced when placental explants were cultured in low glucose medium while the addition of L-lactic acid prevented cell death. In this condition, AQP9 blocking increased the apoptotic indexes. We also confirmed the presence of two mitochondrial subpopulations which exhibit different morphologic and metabolic states. Western blot revealed AQP9 expression only in the heavy/large mitochondrial subpopulation. This is the first report that shows that AQP9 is expressed in the heavy/large mitochondrial subpopulation of trophoblasts. Thus, AQP9 may mediate not only the lactic acid entrance into the cytosol but also into the mitochondria. Consequently, its lack of functionality in preeclamptic placentas may impair lactic acid utilization by the placenta, adversely affecting the survival of the trophoblast cells and enhancing the systemic endothelial dysfunction.

## Introduction

The normal growth and development of the fetus are sustained by the placenta. This ephemeral organ is more than just a selective barrier between the mother and the fetus. It is also a metabolically dynamic interface that uses part of the nutrient uptake to promote its own cellular growth (Vaughan and Fowden, 2016). In this context, emerging evidence shows that the placenta may act as a sensor, detecting the availability of nutrients in the maternal circulation and adapting its metabolism to support fetal development (Díaz et al., 2014; Vaughan and Fowden, 2016).

Glucose is the primary substrate needed to meet the fetus and the placenta energy requirements (Baumann et al., 2002; Hay, 2006). The transfer of glucose across the placenta is mediated by specific Glucose transporters (GLUTs), GLUT1 being the most abundant isoform expressed at term (Illsley, 2000; Baumann et al., 2002). Besides, the level of lactic acid in fetal circulation is higher than in maternal circulation, suggesting that lactic acid could also serve as fuel for the fetus (Vaughan and Fowden, 2016).

Lactic acid exists in two isomeric forms: D-lactic acid and L-lactic acid. However, mammalian cells can only metabolize the L-lactate stereoisomer. Transcellular transfer of lactate is facilitated by a family of transmembrane proteins known as monocarboxylate transport system (MCT) that functions as a proton symport and is stereoselective for L-lactate (Halestrap, 2013; Iwanaga and Kishimoto, 2015). In the brain, it was reported that the transfer of monocarboxylates, such as lactate, may also be facilitated by aquaporin-9 (AQP9) (Badaut, 2010; Tescarollo et al., 2014). It was also found that lactate permeability increases with acidification suggesting that AQP9 may play a role as a channel for the protonated lactic acid form (Rambow et al., 2014). In addition, recent research proposes that lactate can also cross the mitochondrial membranes. In the mitochondria, lactate may be metabolized to pyruvate by the mitochondrial lactate dehydrogenase (LDH), leading to the formation of NADH. Thus, the production of NADH could scavenge reactive oxygen species (ROS) and protect cells from ROS-induced damage (Miki et al., 2013).

AQP9 belongs to a family of integral membrane proteins whose primary role is to facilitate transcellular water fluxes in response to osmotic gradients. In addition to water, AQP9 is also permeable to urea, glycerol, and monocarboxylic acids, like lactic acid, but it is impermeable to cyclic sugars as d-glucose (Tsukaguchi et al., 1998). Unlike MCTs, AQP9 can only transport the protonated form of monocarboxylates (Tsukaguchi et al., 1998; Rothert et al., 2017).

In normal human term placenta, MCT1 and MCT4 are localized on the basal membrane and the apical microvillus membrane of the syncytiotrophoblast cells (Settle et al., 2004; Nagai et al., 2010). MCT4 has a low affinity for lactate, playing a role in lactate export under conditions of high intracellular lactate (Halestrap, 2012). On the other hand, AQP9 is expressed in the apical membrane of the syncytiotrophoblast (Damiano et al., 2001) and the plasma membrane of the cytotrophoblast cells (Wang et al., 2004). However, at term, the cytotrophoblast layer is discontinuous and it does not restrict the transfer between the mother and the fetus.

In several placental disorders, such as preeclampsia, alterations in the formation of the syncytiotrophoblast may change the normal expression and function of many transport proteins and negatively impact the transfer of essential molecules, such as glucose, proteins, and oxygen (Brett et al., 2014).

In this regard, GLUT1 expression and function are downregulated in placentas from preeclamptic women (Lüscher et al., 2017), suggesting a reduction in glucose transport across the placenta. Additionally, a significant decrease was found in aerobic glycolysis in preeclamptic placentas (El-Bacha et al., 2019; Hu et al., 2021). Consequently, the trophoblast cells and the fetus might be driven to use an alternative source of energy like lactate.

Previously, we found that the molecular expression of AQP9 significantly increased in placentas from preeclamptic pregnant women (Damiano et al., 2006). However, functional experiments showed that water and monocarboxylate transport mediated by AQP9 were dramatically reduced (Damiano et al., 2006). Notwithstanding this, there is no evidence of alterations in the transcellular water transport between the mother and the fetus, suggesting that the main role of AQP9 in the human placenta is not related to water transport (Szpilbarg et al., 2018).

Given that AQP9 is also a metabolite channel, we proposed that this protein could be involved in placental energy metabolism. As a result, alterations in AQP9 may enhance syncytiotrophoblast stress, negatively affecting the survival of the cells. This feature may accelerate the release of apoptotic syncytial aggregates into maternal circulation potentially causing the damage of the endothelial cells.

However, the participation of AQP9 in the lactate transfer across the placenta was not investigated yet.

## Materials and Methods

### Tissue Collection

This study was approved by the local ethics committee of the Hospital Nacional Dr. Prof. Alejandro Posadas and the Facultad de Farmacia y Bioquímica, Universidad de Buenos Aires, Argentina [EXP-UBA: 45449/2017 Res(CD) No 2168/2017], and written consent was obtained from patients before the collection of the samples. Full-term normal placentas (*n* = 16) were obtained after cesarean section. All placentas were collected from healthy pregnant women who carried on an uncomplicated pregnancy and gave birth to a newborn without anomalies. Women who carried on multiple pregnancies, and those who had underlying maternal conditions, such as chronic kidney disease, chronic hypertension, liver disease, collagen vascular disease, diabetes, major fetal abnormalities, cardiovascular disease, and cancer, that could adversely affect the pregnancy were excluded. The clinical characteristics of the pregnant women are shown in Table 1.

**Table 1:** Characteristics of pregnant women.

	Normal pregnant women
**Number of pregnant women**	16
**Maternal age**, *yr*	25.6 ± 1.7
**Gestational age**, *wk*	37.5 ± 0.1
**Mean blood pressure**, *mM Hg*	
Systolic	111 ± 3.7
Diastolic	64 ± 1.9
**Proteinuria**	negative
**Body Mass Index (BM)**, *kg/m*^2^	24 ± 3
**Birth weight**, *g*	3450.5 ± 42.8
**Fetal sex**	
Male	9
Female	7
**Placental weight**, *g*	519.4 ± 14.3

Values are mean ± *SD*. All women were white Hispanic.

### Tissue Culture Conditions

The placentas were placed with the maternal side facing up and arbitrarily divided into four quadrants. Cotyledon fragments were isolated from different areas of each placenta midway between the chorionic and basal plate, using sterile dissection. After that, the decidua and basal plate were removed completely, and the placental tissue was thoroughly washed with saline solution to eliminate blood. Villous tissue was further dissected into explants of ∼50 mg and cultured as we previously described (Castro-Parodi et al., 2013). Briefly, explants were preincubated for 30 min in a free-serum medium to allow the tissue to recover from the isolation processes. Then, explants were placed into 24-well plates with low glucose Dulbecco’s modified Eagle’s medium (DMEM, Life Technologies, Inc. BLR, Grand Island, NY, United States) and 100IU/ml penicillin, 100 mg/ml streptomycin, 32 mg/ml gentamicin, and cultured at 37°C during 18 h. This medium contained 5 mM glucose and 1 mM sodium pyruvate, hereafter referred to as the low glucose medium. In some wells, this medium was supplemented with (a) 20 mM glucose (control situation), (b) 10 mM D-Lactic acid (Sigma-Aldrich Corp., San Luis, MO, United States), or (c) 10 mML-Lactic acid (Sigma-Aldrich Corp., San Luis, MO, United States). D-Lactic acid is a stereoisomer of L-Lactic acid, which is not metabolized by mammalian cells. In all situations, osmolarity was adjusted by adding D-mannitol (Sigma-Aldrich Corp., San Luis, MO, United States).

In all the experimental conditions, explants were cultured in the presence and absence of 0.3 mM HgCl_2_ (Sigma-Aldrich, San Louis, MO, United States), a nonselective inhibitor of AQPs, 0.5 mM Phloretin (Sigma-Aldrich, St. Louis, MO, United States) for specific blocking of AQP9 (Inuyama et al., 2002; Haddoub et al., 2009), and 50 mM alpha-cyano-4-hydroxycinnamic acid (CHC, Sigma-Aldrich, St. Louis, MO, United States) (Inuyama et al., 2002), a nonspecific inhibitor of MCTs. HgCl_2_ stock solution was prepared in PBS, while Phloretin and CHC were diluted in DMSO. Vehicle controls were performed and no changes were observed compared with the untreated control (data not shown).

Experiments were conducted independently in triplicates and repeated at least three times.

The protein expression of AQP9 was tested by Western blot in the experimental conditions (Castro-Parodi et al., 2013).

### MTT Incorporation

Viability was assessed by the 3-(4,5-dimethylthiazol-2-yl)-2,5-diphenyl tetrazolium bromide (MTT, Sigma-Aldrich Corp., San Luis, MO, United States) assay as described previously (Castro-Parodi et al., 2013). After treatments, explants were incubated with 0.5 mg/ml MTT for 2 h at 37°C. After this time, each explant was put in another well containing 1 ml methanol to extract the formazan. Optical density was measured at 595 nm and values were relativized to the amount of total protein (Castro-Parodi et al., 2013).

### LDH Release

The release of the cytosolic enzyme LDH in the extracellular environment due to the disruption of the plasma membrane may reflect that cells are dying by necrosis (Chan et al., 2013). LDH release was quantified in the culture medium using the colorimetric method described by Chan and coworkers (Chan et al., 2013). Briefly, LDH catalyzes the oxidation of lactate into pyruvate with the formation of NADH from NAD^+^. Then, NADH is used in the conversion of the tetrazolium salt, 2-p-iodophenyl-3-p-nitrophenyl tetrazolium chloride, into a red formazan product. This reaction is catalyzed by the enzyme diaphorase. Formazan concentrations are directly proportional to the concentration of LDH. The optical density of the formazan product was measured at 492 nm and values were relativized to the amount of total protein.

### Bax/Bcl-2 Ratio

After treatments, placental explants were processed as previously described (Szpilbarg et al., 2016). Briefly, explants were homogenized in lysis buffer containing 0.3M NaCl, 25 mM HEPES, 1.5 mM MgCl_2_, 0.2 mM EDTA, and 1% Triton X-100, pH 7.4, supplemented with protease inhibitors: 0.2 mM PMSF, and 0.01 X Protease Inhibitor Cocktail Set III (Calbiochem^®^, EMD Millipore Corporation, Darmstadt, Germany). After centrifugation at 3100*g* for 10 min, supernatants were collected and protein concentration was estimated by a BCA Protein Assay Kit (Pierce, Thermo Fisher Scientific Inc. Waltham, MA, United States).

80 μg of protein was loaded into each lane, separated on a 12% polyacrylamide gel, and blotted into nitrocellulose membranes (Hybond ECL, Amersham Pharmacia Biotech Ltd., Pittsburgh, PA, United States). Membranes were blocked in 5% nonfat dry milk in Phosphate-buffered saline (PBS, pH 7.5) with 0.1% Tween 20 overnight at 4°C. Subsequently, they were immunoblotted with the primary anti-Bcl-2 (BD Biosciences Pharmingen, NJ, United States; 1:1000) and anti-Bax (Abcam, Cambridge, United Kingdom; 1:200) antibodies.

The antibodies were detected using horseradish peroxidase-linked goat anti-rabbit IgG (Jackson ImmunoResearch Laboratories Inc., West Grove, PA, United States; 1:10,000) or anti-mouse IgG (Jackson ImmunoResearch Laboratories Inc., West Grove, PA, United States; 1:10,000) and visualized by chemiluminescence using the Enhanced Chemiluminescence Western Blotting Analysis System (ECL plus, Amersham Pharmacia Biotech Ltd., Pittsburgh, PA, United States) according to the manufacturer’s instructions. Images were acquired using the ImageQuant LAS 500 chemiluminescence CCD camera (GE Healthcare, CA, United States) and the bands were quantified by the ImageJ 1.45s software package. Detection of Bax and Bcl-2 was performed separately on the same membrane. Equal loading of proteins in each lane was confirmed by staining the blot with Ponceau S (Sigma-Aldrich, San Louis, MO, United States). Results were expressed in arbitrary units.

### Terminal Deoxynucleotidyltransferase-Mediated dUTP Nick-End Labeling Assay

Terminal Deoxynucleotidyltransferase-Mediated dUTP Nick-End Labeling (TUNEL) staining on histological sections was performed to identify apoptotic cells. The *in situ* Cell Death Detection Kit, Tetramethyl rhodamine (TMR) Red (Roche Applied Science, Indianapolis, IN, United States) assay was used according to the manufacturer’s instructions. DAPI counterstaining was used to visualize cell nuclei. Both TMR- (red) and DAPI (blue) fluorescence were visualized by an epifluorescent microscope (Nikon, Eclipse E: 200).

### Isolation of Mitochondrial Fractions and Detection of AQP9

Mitochondrial fraction was obtained from fresh placental tissue as previously described (Bustamante et al., 2014). Briefly, villous tissue free was dissected and homogenized in MSHE buffer (210 mM mannitol, 70 mM sucrose, 1 mM EDTA, and 5 mM Hepes, pH 7.4). The homogenate was centrifuged at 1,500*g* for 10 min. The supernatant was recovered and centrifuged at 11,000*g* for 10 min to sediment the total mitochondrial fraction. The supernatant was ultracentrifuged at 100,000*g* for 60 min, resulting in a pellet designated as the microsomal fraction.

To separate the two subpopulations of mitochondria based on the specified sedimentation velocity, the total mitochondria fraction was centrifuged again at 4,000*g* for 15 min. The obtained pellet corresponds to the “heavy/large” mitochondrial fraction. Following, the supernatant was centrifuged at 12,000*g* for 15 min, obtaining a pellet described as “light/small” mitochondrial fraction (Bustamante et al., 2014). All centrifugations were carried out at 4°C. All the pellets were resuspended in MSHE buffer and protein concentration was determined as described above. Cytosolic and microsomal contaminations were assessed by determination of the specific activities of the lactic dehydrogenase and the antimycin A – insensitive nicotinamide adenine dinucleotide (NADH)-dependent cytochrome C reductase (Ramírez-Vélez et al., 2013; Bustamante et al., 2014).

Proteins were resolved by SDS-PAGE on a 12% gel, electrophoretically transferred to a nitrocellulose membrane. Membrane was probed with a polyclonal anti-AQP9 antibody (Alpha Diagnostic International Inc., San Antonio, TX, United States; 1:1,000) followed by incubation with a goat anti-rabbit immunoglobulin G (IgG; Jackson ImmunoResearch Laboratories Inc., West Grove, PA, United States; 1:10,000) conjugated to peroxidase. Immunoreactivity was detected using the ECL plus (Amersham Pharmacia Biotech Ltd., Pittsburgh, PA, United States) as previously described. To confirm equal loading, each membrane was also stained with Ponceau S as a general protein marker (Lanoix et al., 2012; Szpilbarg and Damiano, 2017).

### Characterization of Placental Mitochondrial Populations

Mitochondrial morphology of each subpopulation was analyzed by flow cytometry using a three-color FAC-SCAN cytometer equipped with a 15-mW air-cooled *λ* = 488-nm argon laser (Becton Dickinson, Franklin Lakes, NJ, United States) (Mattiasson et al., 2003). The mitochondrial size was determined by the Forward angle light scatter (FSC) of photodiode, and the response collected by an E-00 setting with logarithmic amplification gain of 5.39, and the mitochondrial structure was evaluated by the light scattered (SSC) at the perpendicular direction detected by a photomultiplier tube using a voltage of 578 and a linear amplification gain was adjusted to 4.3 (Bustamante et al., 2014).

To study the mitochondrial transmembrane potential (ΔΨm), heavy and light mitochondria fractions were loaded with 30 nM of the potentiometric probe 3,3′-dihexyloxacarbocyanine Iodide (DiOC6, Thermo Fisher Scientific, Waltham, MA, United States) and evaluated by flow cytometry. The isolated mitochondrial fractions were either treated with 200 μm Ca^2+^, to analyze calcium intake handling by mitochondria, 5 μm Carbonyl cyanide 4-(trifluoromethoxy)-phenylhydrazone (FCCP, Sigma-Aldrich, San Louis, MO, United States), as a positive control, or with PBS (control) for additional 5 min and immediately acquired by the cytometer. The working solutions of the probes were diluted in PBS. Fluorescence response was analyzed and differences were quantified in five independent experiments (Bustamante et al., 2014).

### Transmission Electron Microscopy

Each mitochondrial fraction was washed with PBS and fixed in the glutaraldehyde fixation solution 2.5% in PBS for 4 h at 4°C. After washing twice, both fractions were fixed in 1% osmium tetroxide in PBS for 60 min at 4°C.

Subsequently, dehydration of the samples was carried out, using increasing concentrations of alcohol followed by acetone. Then, they were included in a water-soluble epoxy resin, Durcupan (Sigma-Aldrich, San Louis, MO, United States) at 60°C for 72 h to promote the polymerization. Once polymerized, 0.5 μm semi-fine cuts were made using an Ultramicrotome (Reichert Jung Ultracut E). The sections were mounted on slides, stained with toluidine blue, and observed under the light microscope.

The sections obtained in ultramicrotome were mounted on copper grids and were contrasted with uranyl acetate and lead citrate (Reynolds, 1963). Finally, they were observed under a transmission electron microscope (MET Zeiss 109) equipped with a digital camera (Gatan 1,000W). The analysis of each mitochondrial fractions was based on mitochondrial inner membrane topology (Sun et al., 2007).

### Statistical Analysis

The statistical analysis was conducted by GraphPad Prism 7.02 software (GraphPad Software, Inc. La Jolla, CA). All values were expressed as means ± SEM. The significance of the results was analyzed by Student’s *t*-test, one-way ANOVA followed by Bonferroni *post-hoc* test or two-way ANOVA where appropriate. Differences were considered significant at *p* < 0.05.

## Results

### Effect of the Availability of Glucose and Lactate on the Viability of Trophoblast Cells Before and After the Blocking of AQP9

In order to evaluate the use of lactate as a glucose substitute, normal placental explants were cultured in (a) 25 mM glucose medium (control condition), (b) Low glucose medium, (c) Low glucose medium with D-Lactic acid, and (d) Low glucose medium with L-Lactic acid.

**Figure 1:**
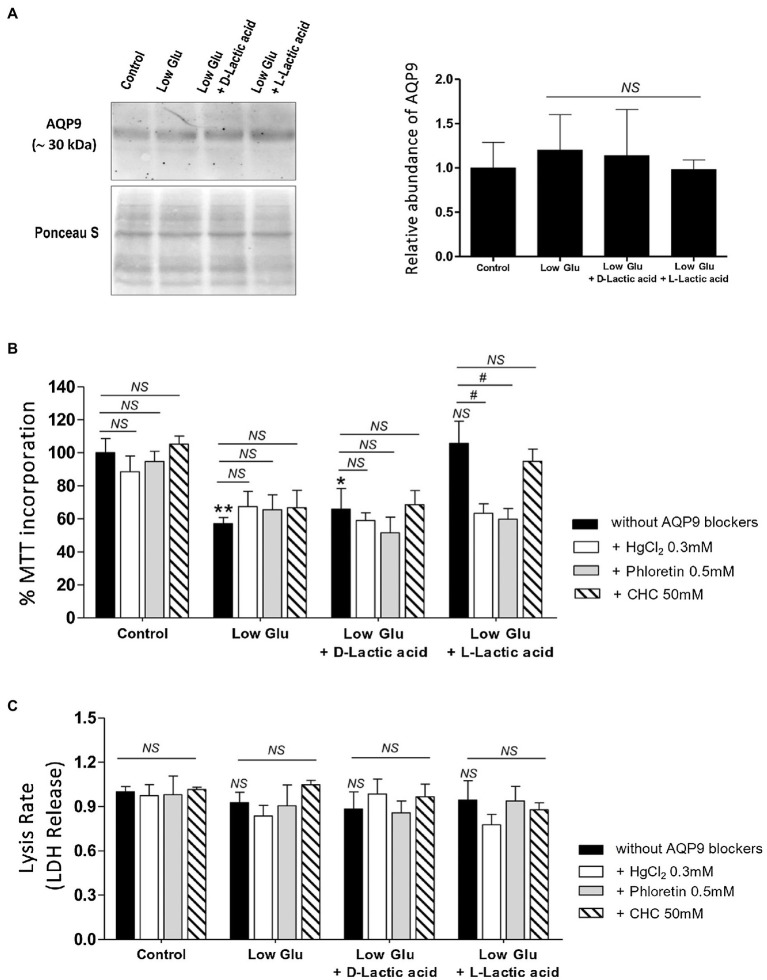
Effect of the availability of glucose and lactate on the expression of Aquaporin-9 (AQP9) and the viability of explants. Explants were cultured in the different conditions: Control (25 mM glucose medium), Low Glucose medium (Low Glu, 5 mM glucose), Low Glucose medium with D-Lactic acid, and Low Glucose medium with L-Lactic acid. **(A)** Expression of AQP9. Representative Western blot for AQP9 protein expression in different culture conditions. Densitometry of immunoblot, the values were plotted as the relative abundance of AQP9 expression. (*n* = 6 placentas; NS = Non-significant). **(B)** 3-(4,5-dimethylthiazol-2-yl)-2,5-diphenyl tetrazolium bromide (MTT) incorporation. The viability was evaluated by MTT assay in placental explants cultured in the different conditions. CHC was used to block MTCs. The effect of 0.5 mM Phloretin or 0.3 mM HgCl_2_ to block AQP9 was evaluated in all the treatments. All the experiments were independently conducted in triplicate. Data are expressed as means ± SEM. (*n* = 6 placentas; ^*^p < 0.05 and ^**^p < 0.001 compared to control without blockers, NS = Non-significant; and ^#^*p* < 0.05 compared to low glucose medium with L-lactic acid without blockers). **(C)** Lactate dehydrogenase (LDH) release. LDH release assay was performed for determining the rate of cell death by necrosis in placental explants cultured in different conditions. CHC was used to block MTCs. The effect of 0.5 mM Phloretin or 0.3 mM HgCl_2_ to block AQP9 was evaluated in all the treatments. The experiments were independently conducted in triplicate at least three times. Data are expressed as means ± SEM. (*n* = 6 placentas; NS = Non-significant). All the experiments were independently conducted in triplicates at least three times.

In all the tested conditions, the protein expression of AQP9 did not change (Figure 1A).

Explant viability, evaluated by MTT incorporation, decreased significantly in low glucose medium even in the presence of D-Lactic acid compared to those explants cultured in the control condition. Interestingly, when explants were cultured in low glucose medium supplemented with L-Lactic acid, MTT levels were similar to control (Figure 1B).

Even more, in this situation, the inhibition of MCTs did not affect cell viability, suggesting that L-lactic acid is passing through another transport protein. To investigate the contribution of AQP9 in the lactic acid transfer, we use HgCl_2_ and Phloretin to block AQP9. We found that in explants cultured in low glucose medium supplemented with L-lactic acid, the blocking with HgCl_2_ and phloretin significantly enhanced cell death. However, no difference was found between both inhibitions. In the other situations, cell death was not modified before and after the blocking of AQP9 (Figure 1B).

In addition, LDH release was analyzed to determine membrane integrity. Membrane leakage is usually associated with cell death by necrosis or late apoptosis. In all the situations tested, the release of LDH into the culture medium did not change, suggesting that cell death is not due to disruption of the plasma membrane (Figure 1C).

### Effect of the Availability of Glucose and Lactate on the Apoptosis of Trophoblast Cells Before and After the Blocking of AQP9

**Figure 2:**
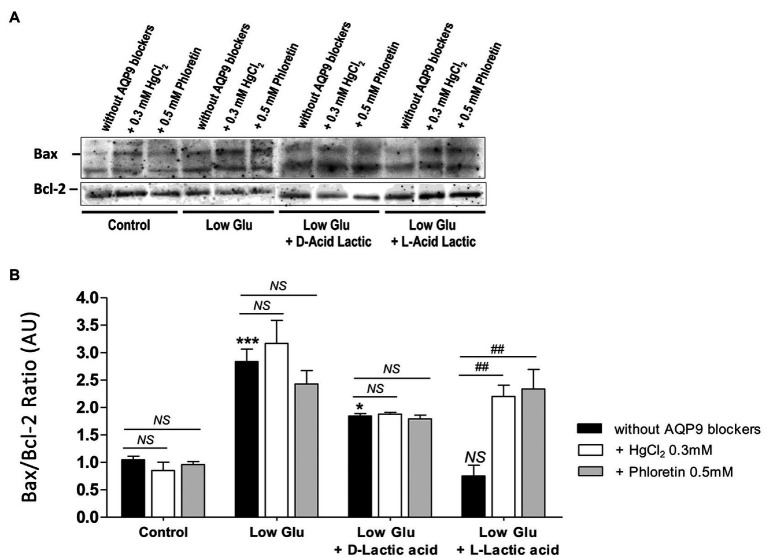
Bax and Bcl-2 expression. **(A)** Representative Western Blot images and **(B)** densitometry for Bax/Bcl-2 ratio in placental explants cultured in the different conditions: Control (25 mM glucose medium), Low Glucose medium (Low Glu, 5 mM glucose), Low Glucose medium with D-Lactic acid, and Low Glucose medium with L-Lactic acid. The effect of 0.5 mM Phloretin or 0.3 mM HgCl_2_ to block AQP9 was evaluated in all the treatments. Data are expressed as means ± SEM. *n* = 6 placentas. (^*^p < 0.05, ^***^p < 0.001 compared to control without AQP9 blockers, NS = Non-significant; and ^##^*p* < 0.01 compared to low glucose medium with L-lactic acid without AQP9 blockers).

**Figure 3:**
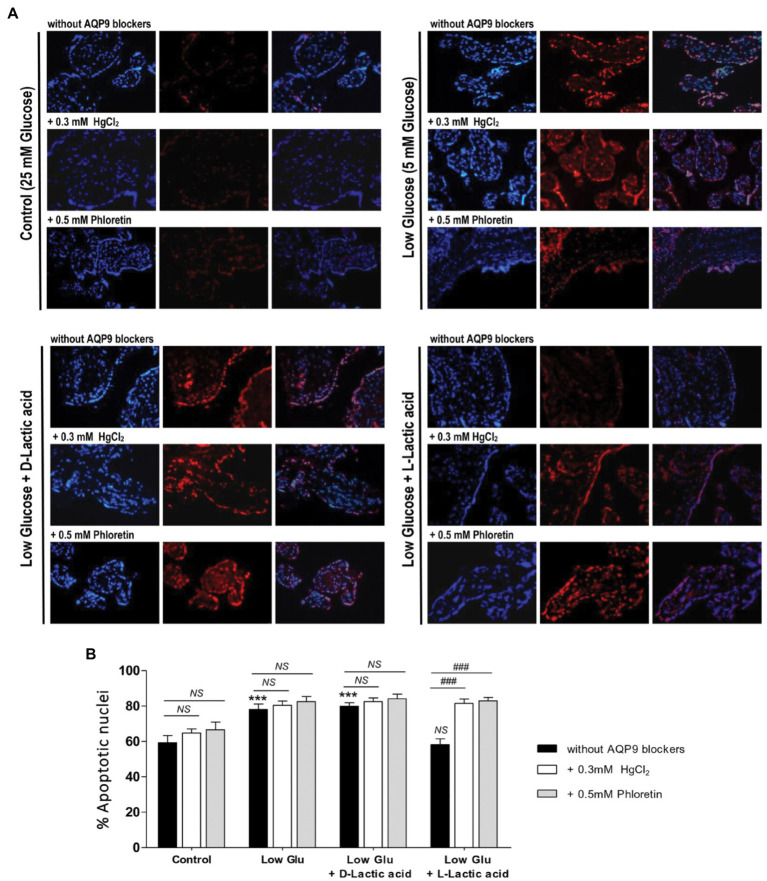
TUNEL assay. **(A)** Representative images and **(B)** % of apoptotic nuclei for each treatment were shown. TUNEL positive cells (red); stroma nuclei stained with DAPI (blue). Image magnification: × 1,000. Data are expressed as means ± SEM. (*n* = 6 placentas; ^***^p < 0.001 compared to control without AQP9 blockers, NS = Non-significant; and ^###^*p* < 0.001 compared to low glucose medium with L-lactic acid without blockers).

To confirm the mechanism of cell death and the participation of AQP9 in the survival of the trophoblast cells, Bax and Bcl-2 expressions and the number of apoptotic nuclei were evaluated. Accordingly with the MTT incorporation, in explants cultured in both low glucose medium and low glucose medium supplemented with D-lactate, Bax/Bcl-2 ratio increased significantly compared to control and remained unaffected after the blocking of AQP9 (Figure 2). The addition of L-lactic acid to the low glucose medium prevented the Bax/Bcl-2 ratio increase (Figure 2). However, in this case, the inhibition of AQP9 resulted in an increased expression of the pro-apoptotic protein Bax and consequently in the Bax/Bcl-2 ratio (Figure 2). Regarding the TUNEL assay, we observed the same behavior. In explants cultured in low glucose medium, the number of TUNEL positive cells was significantly higher than in those cultured in control condition while the addition of L-lactic acid abrogated the apoptosis. As expected, in the medium supplemented with L-lactic acid, the blocking of AQP9 gave rise to an increase in the number of apoptotic nuclei (Figure 3).

### Isolation of Mitochondrial Fractions and Detection of AQP9

To investigate the subcellular localization of AQP9, total mitochondria fraction, and the heavy/large and light/small mitochondria subpopulations that coexist in the villous trophoblasts were isolated by differential centrifugation and characterized by flow cytometry and transmission electron microscopy (TEM). Cytosolic and microsomal contaminations were less than 1.8 and 2.3%, respectively.

In concordance with previous reports, we found that heavy/large particles showed high FSC while the light/small particles presented low FSC, both with similar SSC characteristics suggesting that despite the different sizes of the particles, the internal complexity was similar. The ΔΨm revealed that the light/small mitochondria fraction was depolarized with a level of polarization lower than the heavy/large mitochondrial fraction (Table 2). In the presence of the uncoupler FCCP and 200 μm Ca^2+^, both fractions showed a decrease in their ΔΨm.

**Table 2:** Flow cytometry characterization and mitochondrial membrane potential in isolated mitochondrial fractions.

Mitochondrial Subpopulation	FSC	SSC	ΔΨm		
			Control	FCCP	Ca^2+^ 200 μm
Light/small	13.4 ± 1.8	22.3 ± 1.1	45%	24%	25%
Heavy/large	72.2 ± 1.3^*^	19.2 ± 1.8	64%	34%	29%

* *p* < 0.05 (Heavy/large mitochondrial fraction compared with Light/small mitochondrial fraction).

Values are expressed as means ± SEM, *n* = 5 placentas; % represents the percent respect to the total DIOC6 relative fluorescence intensity quantified in the homogenate. Experiments were independently conducted in triplicates at least three times. FSC = Forward angle light scatter, SSC = Side angle light scatter, ΔΨm = Mitochondrial transmembrane potential, and FCCP = Carbonyl cyanide p-(trifluoromethoxy) phenylhydrazone.

**Figure 4:**
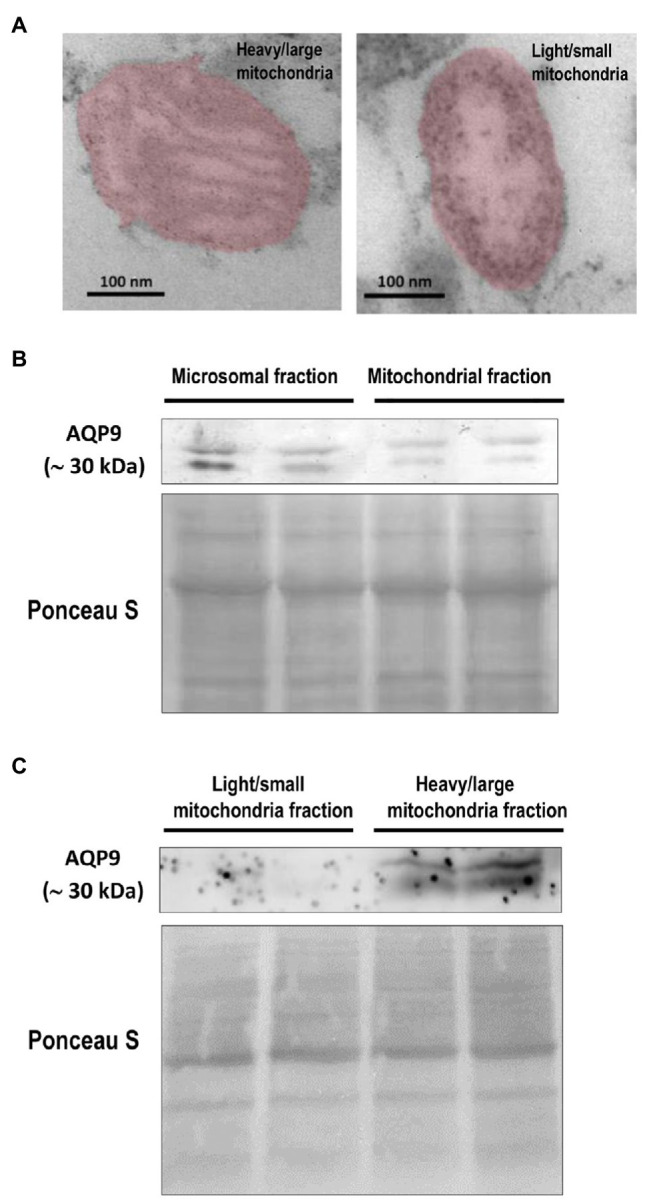
Expression of AQP9 in trophoblast mitochondria. **(A)** Representative transmission electron microscope images of heavy/large and light/small mitochondria found in the isolated subpopulations. Individual mitochondria were falsely colored to aid in identification. The scale bar represents 100 nm and the magnification is 50,000 × (*n* = 4 placentas). **(B)** AQP9 expression in microsomal and mitochondrial fractions. Representative immunoblot revealed that AQP9 is expressed in both mitochondrial and microsomal fractions isolated from villous trophoblast cells. **(C)** AQP9 expression in heavy/large mitochondria subpopulation. Representative immunoblot showed the expression of AQP9 only in the heavy/large fraction (*n* = 12 placentas).

TEM analysis of both mitochondrial fractions confirmed the presence of two phenotypes. Representative TEM micrographs are shown in Figure 4A. The analysis of the mitochondrial phenotype was based mainly on the inner membrane topology. The heavy fraction shows dense staining of the inner matrix with an intact outer membrane showing lamellar cristae. The light mitochondrial fraction exhibits an inner membrane enclosing separate vesicular matrix compartments or cristae and frequently swollen mitochondria with expanded matrix space, lack of staining of the matrix, and fragmented or disorganized phenotype.

Then, we explored the expression of AQP9 in trophoblast mitochondria. We showed evidence for the first time that this protein was found in the mitochondria fraction isolated from villous trophoblast cells (Figure 4B). As we previously reported, a band of 30 kDa corresponding to the AQP9 protein was also found in the microsomal fraction and no band was detected in the nuclear fraction (data not shown). We also analyzed the expression of AQP9 in the heavy and light mitochondrial subpopulations. We found that AQP9 is only present in the heavy mitochondria fraction (Figure 4C).

## Discussion

Previous studies have widely changed the conception that lactate is only a waste metabolic product of cell glycolytic metabolism (Gladden, 2004; Goodwin et al., 2015; Baltazar et al., 2020). In this regard, it is well accepted that glucose is metabolized by the placenta to generate lactic acid, which is the key fuel for fetal growth (Baumann et al., 2002; Hay, 2005, 2006). In sheep, it was reported that lactate produced by the placenta represents almost 25% of fetal oxidative metabolism (Burd et al., 1975). Moreover, an association was found between reduced placental lactate transport to the fetus and fetal growth restriction (Settle et al., 2006). Nevertheless, it was not explored whether the placenta can use lactate to substitute glucose as an energy substrate when its availability in the maternal blood is reduced.

On the other hand, Miki and coworkers have reported that brain AQP9 can work with MCTs to transport lactate and speculated that it could have a role in energy metabolism and/or as a ROS scavenger (Miki et al., 2013; Akashi et al., 2015). Previously, we found that AQP9 expressed in the human placenta may not be only involved in water movement and homeostasis (Castro Parodi et al., 2011; Castro-Parodi et al., 2013). However, the role of AQP9 in the human placenta is still unknown.

In this work, we found that cell death was induced when placental explants were cultured in low glucose medium. There was no evidence of disruption of the plasma membrane, so cell death may take place by apoptosis. In this condition, the addition of L-lactic acid prevented cell death, and interestingly, the inhibition of MCTs did not affect cell viability revealing that another transport protein may be facilitating L-Lactic acid entry into the cell. On the other hand, the blocking of AQP9 led to an increase in both the pro-apoptotic protein Bax and the number of TUNEL positive nuclei in low glucose conditions.

Therefore, our findings suggest that trophoblasts can use L-lactic acid as an alternative source of energy when glucose availability is reduced by an AQP9-mediated mechanism.

It is well established that mitochondria orchestrate the process of life-and-death decisions of the cell (Can et al., 2014; Javadov et al., 2020; Marín et al., 2020).

In many tissues, it was proposed that lactate can enter the mitochondria and be metabolized to pyruvate by the mitochondrial LDH, whereas NAD^+^ is reduced to NADH (Gladden, 2004; Passarella et al., 2014; Goodwin et al., 2015). Thus, NADH generated in the mitochondria can be re-oxidized to NAD^+^ by the electron transport chain, while pyruvate can enter the tricarboxylic acid (TCA) cycle, which allows maintenance of the mitochondrial energy homeostatic cycle (Schurr and Gozal, 2012). Besides, NADH may act as a ROS scavenger. Thus, any alteration in NADH production may give rise to ROS accumulation triggering cell damage and finally leading to cell death (Miki et al., 2013). In this regard, there is considerable evidence that ROS promotes the apoptotic death of villous trophoblasts (Szpilbarg et al., 2016, 2018; Marín et al., 2020).

In the brain, it was reported that AQP9 also localizes in mitochondria (Amiry-Moghaddam et al., 2005), suggesting that mitochondrial AQP9 may function as a monocarboxylate channel working with MCT to transport lactate (Miki et al., 2013; Akashi et al., 2015).

In human placenta, it is well documented that as cytotrophoblast cells differentiate into syncytiotrophoblast cells, trophoblast mitochondria undergo morphological and functional modifications. Previous reports showed that after *in vitro* fusion experiments, an accumulation of numerous small mitochondria was observed in the syncytial cells (Martinez et al., 1997). Thus, the “heavy” mitochondria fraction may be related to the cytotrophoblast while the “light” fraction may be linked to the syncytiotrophoblast (Martinez et al., 1997; Bustamante et al., 2014; Fisher et al., 2020). However, both mitochondria subpopulations may coexist in the syncytiotrophoblast.

In this context, we isolated both fractions and explored the expression of AQP9 in trophoblast mitochondria. According to previous work, we confirmed that the light/small mitochondria subpopulation is less polarized than the heavy one (Bustamante et al., 2014). Furthermore, the electron microscopy images showed well-defined differences not only in the mitochondrial morphology of each subpopulation but also in the inner membrane topology. Our results also revealed that AQP9 is present in the villous trophoblast mitochondria. Even more, this is the first report that shows evidence that this protein was only observed in the large/heavy mitochondria subpopulation.

Bustamante and coworkers have reported that the “heavy” fraction showed a better respiratory function, lower hydrogen peroxide production, lower mitochondrial P450, and higher cardiolipin concentration than the “light” fraction. In addition, they demonstrated that the “heavy” fraction expressed significant protein levels of p53, Bax, and cytochrome c compared with the “light” fraction (Bustamante et al., 2014). Based on these data, they suggested that the reduced oxygen consumption capacity, observed in the light fraction, may be related to a decrease in ATP production (Bustamante et al., 2014). Besides generating ATP, mitochondria also serve as local calcium (Ca^2+^) buffers that tightly regulate intracellular Ca^2+^ levels (Haché et al., 2011). In this way, the electrochemical potential across mitochondria’s inner membrane is used to sequester Ca^2+^. Thus, a lower ΔΨm in the small/light fraction may reflect that calcium ions are dissipated more slowly across the inner mitochondrial membrane into the mitochondrial matrix, affecting the speed of electron transfer *via* the oxidative phosphorylation complexes and the citric acid cycle activity (Bertero and Maack, 2018).

All these differences suggest that both mitochondria fractions could be involved in different cellular processes. In this regard, Fisher and coworkers have recently proposed that the heavy mitochondrial subpopulation may participate in the physiological apoptotic mechanisms required for the normal differentiation and turnover of villous trophoblast cells (Fisher et al., 2020). Meanwhile, the light fraction may execute necrosis or autophagy (Fisher et al., 2020).

**Figure 5:**
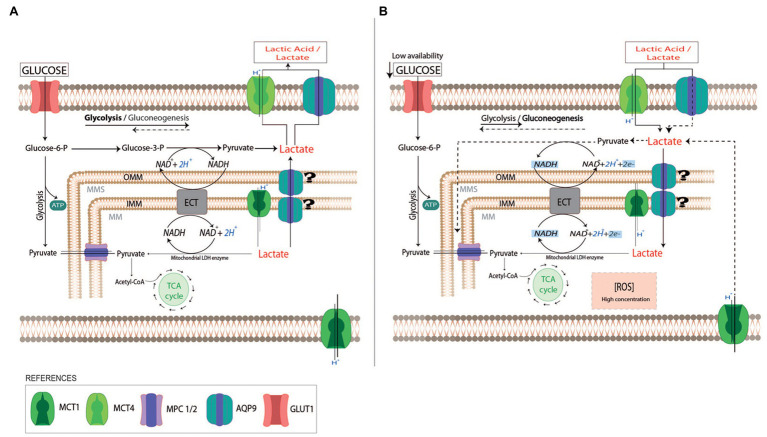
Schematic representation of lactate use by trophoblast cells. In normal pregnancies, trophoblast cells use glucose as a fuel to support the placenta’s cellular growth (A). The excess of lactate due to fetal metabolism may be driven into the maternal circulation by monocarboxylate transports system (MCTs) and AQP9. When the availability of glucose is reduced (B), trophoblast cells can use lactate as a carbon source substitute (dot lines). The uptake of lactate into the cytosol may be facilitated by AQP9 and MCTs localized in the plasma membrane. In the cytosol, lactate can be metabolized into pyruvate or it can pass across the external and inner mitochondria membranes by MCTs or AQP9. In the mitochondria matrix, lactate may be oxidated to pyruvate by a mitochondrial LDH while NAD^+^ is reduced to NADH. The produced NADH may act as a reactive oxygen species scavenger. In preeclamptic placentas, AQP9 is not functional altering the use of lactate by the trophoblast cells. This may affect the mitochondria function, leading to the activation of the mitochondrial pathway of apoptosis. OMM, Outer Mitochondria membrane; IMM, Inner Mitochondria membrane; ECT, Electron transport chain; TCA cycle, tricarboxylic acid cycle; and MPC 1/2, Mitochondrial pyruvate carrier 1 and 2.

The evidence presented here supports the idea that in trophoblast cells, AQP9 may function as a lactate transporter together with MCTs. Since we found that AQP9 localized not only in the apical membrane (Damiano et al., 2001) but also in the mitochondria of the villous trophoblast cells, this protein may facilitate not only the lactic acid entrance into the cytosol but also into the mitochondria (Figure 5A). Along with this, we found that in a reduced glucose medium supplemented with L-Lactic acid, lactic acid cannot enter the cell when AQP9 is blocked, impairing mitochondrial function, resulting in the activation of the mitochondrial pathway of apoptosis. Therefore, it is possible that the ability of the villous trophoblast cells to better respond to the stress may be related to the content of heavy/large mitochondria with a functional AQP9.

It is well accepted that preeclampsia is usually associated with intermittent placental perfusion. Consequently, fluctuations in O_2_ tension may enhance placental oxidative stress which has a critical role in exacerbating the villous trophoblast apoptosis (Hung et al., 2002; Hung and Burton, 2006; Marín et al., 2020). Considering the reduced GLUT1 expression and the decreased aerobic glycolysis observed in preeclamptic placentas, the concentrations of lactate in the placenta and the maternal blood might be increased. Although several reports have shown that plasma lactate levels are high in preeclampsia (Peguero et al., 2019), lactate concentrations are low in the placentas from preeclamptic women, suggesting that lactate cannot pass across the cell membrane of the trophoblasts.

Accordingly, we speculated that the increased oxidative stress observed in preeclampsia may impair AQP9 function as a lactate transporter. In this scenario, the lack of functionality of AQP9 may impair the lactic acid utilization by the placenta, promoting more accumulation of ROS and adversely affecting the survival of the trophoblast cells. This stress in the trophoblast cells may enhance the shedding of apoptotic aggregates into maternal circulation resulting in the systemic endothelial dysfunction that characterizes the maternal syndrome. Therefore, a non-functional AQP9 might be involved in the pathogenesis of preeclampsia.

## Data Availability Statement

The raw data supporting the conclusions of this article will be made available by the authors, without undue reservation.

## Ethics Statement

The studies involving human participants were reviewed and approved by Hospital Nacional Dr. Prof. Alejandro Posadas and Facultad de Farmacia y Bioquímica, Universidad de Buenos Aires, Argentina [EXP-UBA: 45449/2017 Res(CD) N2168/2017]. The patients/participants provided their written informed consent to participate in this study.

## Author Contributions

YM, LA, and JR carried out the experimental work and analysis of data. AC provided the placental tissues and discussed the results. NS and JB carried out data analysis and discussion and critically reviewed the manuscript. AD designed the study and wrote the manuscript. All authors contributed to the final version of the manuscript.

## Funding

This study was supported by UBACyT 2018 (20020170100194BA) and ANCyPT (PICT 2018-02322) grants.

## Conflict of Interest

The authors declare that the research was conducted in the absence of any commercial or financial relationships that could be construed as a potential conflict of interest.

## Publisher’s Note

All claims expressed in this article are solely those of the authors and do not necessarily represent those of their affiliated organizations, or those of the publisher, the editors and the reviewers. Any product that may be evaluated in this article, or claim that may be made by its manufacturer, is not guaranteed or endorsed by the publisher.
